# Proton Pump Inhibitors and the Risk of Adverse Cardiac Events 

**DOI:** 10.1371/journal.pone.0084890

**Published:** 2013-12-27

**Authors:** David N. Juurlink, Colin R. Dormuth, Anjie Huang, Chelsea Hellings, J. Michael Paterson, Colette Raymond, Anita Kozyrskyj, Yola Moride, Erin M. Macdonald, Muhammad M. Mamdani

**Affiliations:** 1 The Institute for Clinical Evaluative Sciences, Toronto, Ontario, Canada; 2 The Sunnybrook Research Institute, Toronto, Ontario, Canada; 3 University of British Columbia, Vancouver, British Columbia, Canada; 4 Keenan Research Centre of the Li Ka Shing Knowledge Institute, St. Michael’s Hospital, Toronto, Ontario, Canada; 5 Department of Medicine, University of Toronto, Toronto, Ontario, Canada; 6 Department of Health Policy, Management, and Evaluation, University of Toronto, Toronto, Ontario, Canada; 7 Leslie Dan Faculty of Pharmacy, University of Toronto, Toronto, Ontario, Canada; 8 Department of Family Medicine, McMaster University, Hamilton, Ontario, Canada; 9 Centre for Evaluation of Medicines, St. Joseph’s Healthcare, Hamilton, Ontario, Canada; 10 Manitoba Centre for Health Policy, Faculty of Medicine, University of Manitoba, Winnipeg, Manitoba, Canada; 11 The Winnipeg Regional Health Authority, Winnipeg, Manitoba, Canada; 12 Women and Children’s Research Institute and the Department of Pediatrics, Faculty of Medicine & Dentistry, University of Alberta, Calgary, Alberta, Canada; 13 Faculty of Pharmacy, Université de Montréal, Montréal, Quebec, Canada; Virginia Commonwealth University, United States of America

## Abstract

**Background:**

Recent evidence suggests that proton pump inhibitors (PPIs) might be linked with adverse cardiac events, but a causal relationship is unproven.

**Methods:**

We applied the self-matched case series method to two studies using population-based health care data from Ontario, Canada between 1996 and 2008. The first included subjects aged 66 years or older hospitalized for acute myocardial infarction within 12 weeks following initiation of PPI, while the second included subjects hospitalized for heart failure. In both studies we designated the primary risk interval as the initial 4 weeks of therapy and the control interval as the final 4 weeks. To test the specificity of our findings we examined use of histamine H2 receptor antagonists and benzodiazepines, drugs with no plausible causal link to adverse cardiac events.

**Results:**

During the 13-year study period, we identified 5550 hospital admissions for acute myocardial infarction and 6003 admissions for heart failure within 12 weeks of commencing PPI therapy. In the main analyses, we found that initiation of a PPI was associated with a higher risk of acute myocardial infarction (odds ratio 1.8; 95% confidence interval 1.7 to 1.9) and heart failure (odds ratio 1.8; 95% confidence interval 1.7 to 1.9). However, secondary analyses revealed similar risk estimates histamine H2 receptor antagonists and benzodiazepines, drugs with no known or suspected association with adverse cardiac events.

**Conclusion:**

PPIs are associated with a short-term risk of adverse cardiac events, but similar associations are seen with other drugs exhibiting no known cardiac toxicity. Collectively these observations suggest that the association between PPIs and adverse cardiac events does not represent reflect cause-and-effect.

## Introduction

Proton pump inhibitors (PPIs) are among the most widely prescribed medications in North America, with up to a third of older patients in some jurisdictions taking these drugs for treatment of peptic ulcer disease, gastroesophageal reflux or prevention of NSAID gastropathy [1,2]. Although these drugs are generally perceived to be safe, recent reports suggest they may be risk factors for interstitial nephritis, osteoporosis and *Clostridium difficile*–associated disease [3-5]. 

A small number of observational studies have also suggested that PPIs might be independently associated with adverse cardiac events [3,4]. A post-hoc analysis of the PLATelet inhibition and patient Outcomes (PLATO) trial reached similar conclusions [5], although the investigators conducted several supplemental analyses suggesting that the findings were likely to reflect bias or confounding. Indeed, there is little biologic plausibility to support the notion that PPIs might directly cause adverse cardiac events. One *ex vivo* study found that physiologic concentrations of pantoprazole impaired myocardial contraction in human and rabbit cardiac muscle in a dose-dependent fashion [6], but healthy volunteers display no overt impairment in left ventricular systolic function following administration of the drug intravenously [7]. 

Using population-based healthcare databases, we examined the potential association between PPI use and hospitalization for acute myocardial infarction (AMI) or heart failure (HF).

## Methods

### Ethics Statement

The study was approved by the institutional review board at Sunnybrook Health Sciences Centre, Toronto, Ontario. For the purposes of this research informed consent was not required. The Institute for Clinical Evaluative Sciences (ICES) is named as a prescribed entity in Section 45 of the *Personal Health Information Protection Act* (PHIPA - Regulation 329/04, Section 18). Under this designation, ICES can receive and use health information without consent for purposes of analysis and compiling statistical information about the Ontario health care system.

### Study Design

We used the self-matched case-series method described by Farrington8 to explore the temporal association between initiation of PPI therapy and adverse cardiac events (AMI or HF) among Ontario residents aged 66 years and older from January 1, 1996 to December 31, 2008. This approach is increasingly used to explore short-term adverse effects of drug exposure, and is conditional on both exposure and occurrence of the adverse outcome of interest within a predefined period. A major advantage of this design is that patients serve as their own controls, implicitly controlling for fixed patient factors and thereby eliminating unmeasured confounding that can sometimes threaten the validity of case-control and cohort studies.

### Data Sources

Prescription drug records were obtained from the Ontario Drug Benefit Claims Database, and information on hospital admissions was collected using the Canadian Institute for Health Information’s Discharge Abstract Database (CIHI-DAD). Demographic information was derived from the Registered Persons Database, which contains an entry for each resident of Ontario who has been issued a health card. Finally, the Ontario Health Insurance Plan Database provided information regarding claims for physician services. These databases are linked anonymously using encrypted health card numbers, and are routinely used to study drug safety [9-11]. For most common cardiovascular diagnoses, the coding quality in the CIHI-DAD is very good to excellent.

### Assessment of Exposure and Outcome

We defined the index date as the date of a first prescription for a PPI. We examined the risk of hospitalization for all patients hospitalized for AMI (International Classification of Disease (ICD-9) codes 410, 411, 414 or ICD-10 codes I21, I240, I241, I248, I249, I254) or HF (ICD-9 code 428 and ICD-10 code I50). These codes have been validated previously [12-15]. For patients hospitalized with AMI, we excluded those discharged within 3 days under the assumption that a true AMI was unlikely [16]. 

In keeping with the self-matched case series design, we included only those hospitalizations occurring within 12 weeks of initiation of PPI treatment, reasoning that susceptible patients would manifest adverse effects shortly after the start of treatment. For the primary analysis, we excluded patients with a previous hospitalization for AMI or HF within one year preceding the index date. Because AMI and HF are both associated with substantial mortality, we conducted secondary analyses limited to patients who were alive at the end of the 12-week follow-up period.

Additional analyses examined the risk of hospitalization for a cardiac event among patients who had a history of AMI or HF, as they are likely to be at increased risk. For this analysis, we considered a patient to have a history of the condition if there was a hospitalization for either AMI or HF in the 6 to 12 month period preceding the initiation of a PPI. (For this supplementary analysis, we did not include patients hospitalized in the 6 months prior to the start of PPI therapy, because this would have interfered with our ability to reliably ascertain the timing of PPI initiation.) 

To test the robustness and specificity of our findings, we conducted several additional analyses. We performed “tracer” analyses using prescriptions for histamine H2 receptor antagonists and benzodiazepines. Neither of these drug classes has a plausible causal link to adverse cardiac events, and we reasoned that a null finding with these drugs would enhance the argument for a cause-and-effect relationship in our main analysis. Finally, we replicated all analyses using risk and reference intervals of two weeks duration rather than four, separated by a washout period of two weeks. 

### Statistical Analysis

**Figure 1:**
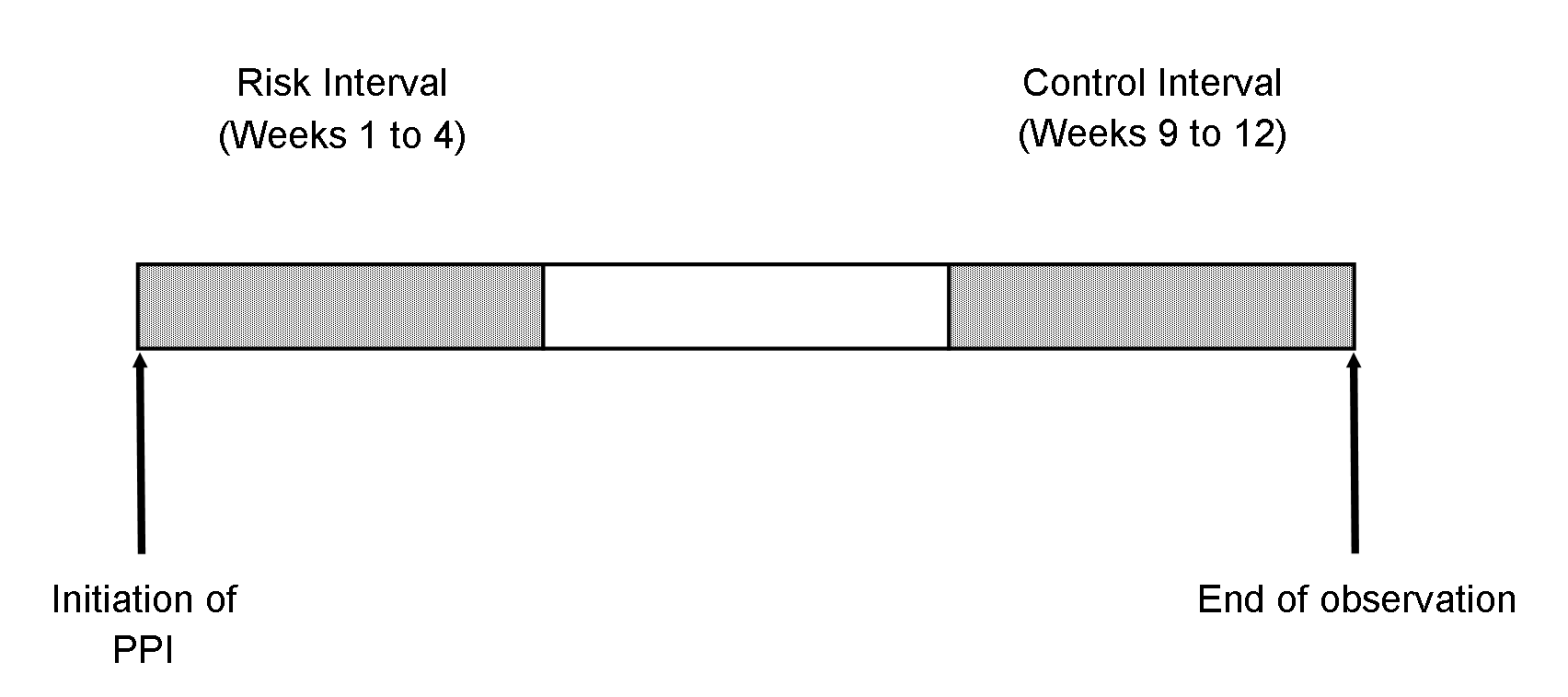
Study Design. The observation period for each patient begins with initiation of a proton pump inhibitor (PPI) and continues for 12 weeks. All patients were admitted to hospital for an acute myocardial infarction or heart failure at some point during the 12-week observation period, which for analytical purposes is divided into three identical 4-week intervals. The first of these is the risk interval, and the final interval defined the control interval.

For analytical purposes, we divided each patient’s follow-up into three identical 4-week intervals. The first 4-week period following initiation of a PPI was considered the primary risk interval (Figure 1), during which time admissions for AMI or HF might reflect an unintended consequence of drug therapy. The final 4-week interval defined the control interval; its remoteness from the exposure renders a causal association with drug therapy highly unlikely. The odds ratio of AMI or HF during the risk period compared to the control period was estimated using a fixed-effects logistic regression model that included exposure and control period terms, and an indicator variable for each patient that allowed each individual to serve as his or her own control. These analyses were replicated using a random effects logistic regression model. All analyses were performed using SAS version 9.2 (SAS Institute, Cary, North Carolina)

## Results

Over the 13-year study period, we identified 5550 hospitalizations for AMI within 12 weeks of the initiation of a PPI. The median age of these patients was 77 years (interquartile range 72 to 82), 49% were female, and 956 (17.2%) died during the 12-week observation period. In the primary analysis, the estimated odds ratio of hospitalization due to AMI during the risk interval compared with the control interval was 1.8 (95% confidence interval 1.7 to 1.9) (Table 1). The risk was slightly accentuated among patients with any history of AMI (estimated odds ratio 2.1; 95% confidence interval 1.6 to 2.7) 

**Table 1:** Risk of hospitalization for an adverse cardiac event following the initiation of a proton pump inhibitor.

**Analysis**	**Admissions for Cardiac Event during Risk Interval (N)**	**Admissions for Cardiac Event during Control Interval (N)**	**Odds Ratio (95% CI)**
**Primary**			
AMI	2595	1439	1.8 (1.7 to 1.9)
HF	2713	1534	1.8 (1.7 to 1.9)
**Secondary**			
AMI (excluding deaths)*	2039	1316	1.5 (1.4 to 1.7)
HF (excluding deaths)*	1985	1378	1.4 (1.3 to 1.5)
History of AMI	175	85	2.1 (1.6 to 2.7)
History of HF	204	116	1.8 (1.4 to 2.2)

* Secondary analysis excluded all deaths within the 12-week observation period

We also identified 6003 subjects hospitalized for HF within 12 weeks of initiation of a PPI. The mean age of these patients was 80 years (interquartile range 74 to 85), 55% were female, and 1235 (20.6%) died during follow-up. In the primary analysis, the estimated odds ratio of HF during the first 4 weeks following initiation of a PPI was 1.8 (95% confidence interval 1.7 to 1.9) (Table 1). The risk was similar among patients with a history of HF (estimated odds ratio 1.8; 95% confidence interval 1.4 to 2.2). 

We found similar results when we examined the risk of hospitalization for AMI and HF in patients without history of these conditions within 12 weeks of the initiation of histamine H2 receptor antagonists (estimated odds ratio 1.8, 95% CI, 1.7 to 1.9 and 1.5, 95% CI, 1.4 to 1.6, respectively) or benzodiazepines (estimated odds ratio 1.3, 95% CI 1.3 to 1.4 and 1.6, 95% CI 1.5 to 1.7, respectively) (both analyses shown in Table 2). 

**Table 2:** Risk of hospitalization for an adverse cardiac event following the initiation of H2 receptor antagonists or benzodiazepines.

**Analysis**	**Admissions for Cardiac Event during Risk Interval (N)**	**Admissions for Cardiac Event during Control Interval (N)**	**Odds Ratio (95% CI)**
**H2 receptor antagonists**			
AMI (excluding deaths)	2384	1336	1.8 (1.7 to 1.9)
HF (excluding deaths)	1910	1287	1.5 (1.4 to 1.6)
**Benzodiazepines**			
AMI (excluding deaths)	2100	1569	1.3 (1.3 to 1.4)
HF (excluding deaths)	2782	1760	1.6 (1.5 to 1.7)

Because some PPIs – omeprazole in particular - can interfere with the bioactivation of clopidogrel [17,18], we performed several *post-hoc* sensitivity analyses to examine the specificity of our findings. The association between omeprazole use and MI or heart failure (odds ratio 1.6; 95% confidence interval 1.6 to 2.0 for both analyses) was no different than that seen with pantoprazole, which does not alter the response to clopidogrel (AMI odds ratio 1.7; 95% confidence interval 1.4 to 2.0; HF odds ratio 1.7; 95% confidence interval 1.5 to 2.0). And while only 283 subjects in our sample were taking clopidogrel at the time they commenced a PPI, these individuals had no differential risk of AMI (odds ratio 1.6; 95% confidence interval 1.0 to 2.5) or HF (odds ratio 2.2; 95% confidence interval 1.5 to 3.3) relative to patients not taking clopidogrel (odds ratio 1.8; 95% confidence interval 1.7 to 1.9 for both MI and HF).

To test the robustness of our conclusions, we replicated our analyses using a random effects model, which tends to yield less precise estimates relative to fixed effects models. In each instance, the conclusions generated in our primary analysis held (data not shown). Finally, we replicated our analyses using risk, washout and control periods of two weeks each rather than four weeks. In each instance, the results were consistent with our main analysis (Table S1, Table S2, Table S3, Table S4).

## Discussion

Using population-based healthcare records over a 13-year period, we found a nearly two-fold higher risk of hospitalization for AMI or HF following the initiation of a PPI in a large cohort of older Ontarians. These findings accord with other lines of evidence suggesting an association between PPI therapy and cardiac events. However, we also found similar risks with histamine H2 receptor antagonists and benzodiazepines, drugs with no plausible causal link to adverse cardiac events. Collectively, these findings imply that cause-and-effect is an unlikely explanation for the observed association between PPIs and adverse cardiac events.

Protopathic bias may partially explain the observed association between PPIs and adverse cardiac events observed in our study. These drugs are often used to treat peptic ulcer disease and esophagitis, conditions that can cause symptoms that may be confused with those of cardiac ischemia. While this is also true of H2 antagonist therapy, it is less likely to explain the observed association between benzodiazepines and adverse cardiac events. Another important limitation of our study is that we restricted the risk period to the first 4 weeks following the initiation of a PPI, reasoning that this would facilitate the detection of any safety signal if one existed, and also because the often-intermittent nature of PPI therapy would render studies of longer-term follow-up less reliable. Finally, PPIs may be used sporadically, particularly in patients with gastroesophageal reflux. However, this would tend to attenuate any effects in our analyses.

Our study has several notable strengths. We utilized more than a decade of population-based hospital records, studying patients in real-world practice. We employed a self-matched design, implicitly controlling for fixed patient characteristics, unlike other observational designs that are more susceptible to selection bias and unmeasured confounding. Finally, we conducted several sensitivity analyses using other medications, all of which yielded similar results. Some limitations also merit emphasis, including a lack of information on drug dose and adherence, as well as risk factors for cardiovascular disease including obesity and smoking. However, the importance of these limitations is lessened by the self-matched nature of the design. 

In summary, in a large population-based study, we found that initiation of PPI therapy was associated with a short-term risk of AMI and HF. However, a risk of similar magnitude was seen with other drugs not suspected of exerting cardiac toxicity, suggesting that the association identified with PPIs is spurious and does not reflect cause-and-effect. These findings should reassure patients and clinicians that use of PPIs when clinically indicated is not associated with adverse cardiac events, even in patients with a history of cardiac disease.

## Supporting Information

Table S1 **Hospitalization for adverse cardiac events within two weeks of initiation of a proton pump inhibitor (fixed effects logistic regression model).**
(DOCX)

Table S2 **Hospitalization for adverse cardiac events within two weeks of initiation of a H2 receptor antagonist or benzodiazepine (fixed effects logistic regression model).**
(DOCX)

Table S3 **Hospitalization for adverse cardiac events within two weeks of initiation of a proton pump inhibitor (random effects logistic regression model).**
(DOCX)

Table S4 **Risk of hospitalization for an adverse cardiac event following the initiation of a H2 receptor antagonist or benzodiazepine (random effects logistic regression).**
(DOCX)
