# Supplementary material for: Proton Pump Inhibitors and the Risk of Adverse Cardiac Events
Source: PLoS One. 2013 Dec 27;8(12):e84890. doi: 10.1371/journal.pone.0084890 (PMC3873988; doi:10.1371/journal.pone.0084890)
Supplement: Table S1 — Hospitalization for adverse cardiac events within two weeks of initiation of a proton pump inhibitor (fixed effects logistic regression model). (DOCX) [file pone.0084890.s001.docx]

**Table S1: Hospitalization for adverse cardiac events within two weeks of initiation of a proton pump inhibitor (fixed effects logistic regression model)**

| **Analysis** | **Admissions for Cardiac Event during Risk Interval (N)** | | **Admissions for Cardiac Event during Control Interval (N)** | **Odds ratio (95% CI)** |
| --- | --- | --- | --- | --- |
| **Primary** | | | | |
| AMI | 1639 | 805 | | 2.0 (1.9 to 2.2) |
| HF | 1606 | 950 | | 1.7 (1.6 to 1.8) |
| **Secondary** | | | | |
| AMI (excluding deaths)* | 1350 | 748 | | 1.8 (1.7 to 2.0) |
| HF (excluding deaths)* | 1288 | 896 | | 1.4 (1.3 to 1.6) |
| History of AMI | 94 | 63 | | 1.5 (1.0 to 2.0) |
| History of HF | 124 | 73 | | 1.7 (1.3 to 2.3) |

*Secondary analysis excluded all deaths within the 6-week observation period
