# Supplementary material for: Proton Pump Inhibitors and the Risk of Adverse Cardiac Events
Source: PLoS One. 2013 Dec 27;8(12):e84890. doi: 10.1371/journal.pone.0084890 (PMC3873988; doi:10.1371/journal.pone.0084890)
Supplement: Table S2 — Hospitalization for adverse cardiac events within two weeks of initiation of a H2 receptor antagonist or benzodiazepine (fixed effects logistic regression model). (DOCX) [file pone.0084890.s002.docx]

**Table S2: Hospitalization for adverse cardiac events within two weeks of initiation of a H2 receptor antagonist or benzodiazepine (fixed effects logistic regression model)**

| **Analysis** | **Admissions for Cardiac Event during Risk Interval (N)** | | **Admissions for Cardiac Event during Control Interval (N)** | **Odds ratio (95% CI)** |
| --- | --- | --- | --- | --- |
| **H2 receptor antagonists** | | | | |
| AMI (excluding deaths) | 1629 | 843 | | 1.9 (1.8 to 2.1) |
| HF (excluding deaths) | 1264 | 805 | | 1.6 (1.4 to 1.7) |
| **Benzodiazepines** | | | | |
| AMI (excluding deaths) | 1367 | 988 | | 1.4 (1.3 to 1.5) |
| HF (excluding deaths) | 1923 | 1233 | | 1.6 (1.5 to 1.7) |
