# Supplementary material for: Proton Pump Inhibitors and the Risk of Adverse Cardiac Events
Source: PLoS One. 2013 Dec 27;8(12):e84890. doi: 10.1371/journal.pone.0084890 (PMC3873988; doi:10.1371/journal.pone.0084890)
Supplement: Table S4 — Risk of hospitalization for an adverse cardiac event following the initiation of a H2 receptor antagonist or benzodiazepine (random effects logistic regression). (DOCX) [file pone.0084890.s004.docx]

**Table S4: Risk of hospitalization for an adverse cardiac event following the initiation of a H2 receptor antagonist or benzodiazepine (random effects logistic regression)**

| **Analysis** | **Admissions for Cardiac Event during Risk Interval (N)** | | **Admissions for Cardiac Event during Control Interval (N)** | **Odds ratio (95% CI)** |
| --- | --- | --- | --- | --- |
| **H2 receptor antagonists** | | | | |
| AMI (excluding deaths) | 1629 | 843 | | 2.8 (2.5 to 3.1) |
| HF (excluding deaths) | 1264 | 805 | | 2.0 (1.8 to 2.2) |
| **Benzodiazepines** | | | | |
| AMI (excluding deaths) | 1367 | 988 | | 1.7 (1.5 to 1.8) |
| HF (excluding deaths) | 1923 | 1233 | | 2.0 (1.8 to 2.2) |
